# Implantable Drug Delivery Systems and Foreign Body Reaction: Traversing the Current Clinical Landscape

**DOI:** 10.3390/bioengineering8120205

**Published:** 2021-12-09

**Authors:** Alexey Fayzullin, Alesia Bakulina, Karen Mikaelyan, Anatoly Shekhter, Anna Guller

**Affiliations:** 1 Department of Experimental Morphology and Biobanking, Institute for Regenerative Medicine, Sechenov First Moscow State Medical University (Sechenov University), 119991 Moscow, Russia; fayzullin_a_l@staff.sechenov.ru (A.F.); bakulina_a_a@staff.sechenov.ru (A.B.); mikaelyan_k_a@staff.sechenov.ru (K.M.); shekhter_a_b@staff.sechenov.ru (A.S.); 2 World-Class Research Center “Digital Biodesign and Personalized Healthcare”, Sechenov First Moscow State Medical University (Sechenov University), 119991 Moscow, Russia; 3 Faculty of Medicine, Health and Human Sciences, Macquarie University, Sydney, NSW 2109, Australia; 4 Biomolecular Discovery Research Centre, Macquarie University, Sydney, NSW 2109, Australia

**Keywords:** implantable drug delivery systems, foreign body reaction, peri-implantitis, fibrosis, scarring, clinical applications, translation, drug repurposing

## Abstract

Precise delivery of therapeutics to the target structures is essential for treatment efficiency and safety. Drug administration via conventional routes requires overcoming multiple transport barriers to achieve and maintain the local drug concentration and commonly results in unwanted off-target effects. Patients’ compliance with the treatment schedule remains another challenge. Implantable drug delivery systems (IDDSs) provide a way to solve these problems. IDDSs are bioengineering devices surgically placed inside the patient’s tissues to avoid first-pass metabolism and reduce the systemic toxicity of the drug by eluting the therapeutic payload in the vicinity of the target tissues. IDDSs present an impressive example of successful translation of the research and engineering findings to the patient’s bedside. It is envisaged that the IDDS technologies will grow exponentially in the coming years. However, to pave the way for this progress, it is essential to learn lessons from the past and present of IDDSs clinical applications. The efficiency and safety of the drug-eluting implants depend on the interactions between the device and the hosting tissues. In this review, we address this need and analyze the clinical landscape of the FDA-approved IDDSs applications in the context of the foreign body reaction, a key aspect of implant–tissue integration.

## 1. Introduction

Controlled and target-specific drug delivery is of critical importance in modern medicine. Therapeutic windows of conventional oral and intravenous drug administration routes are often limited by the undesirable side effects on non-target tissues and suboptimal first-pass metabolism [1]. Moreover, the complex routines of patients who need to take multiple pills or make injections hinder the medication through the low adherence that results in unstable therapeutic concentrations [2,3]. In the drug development field, systemic side effects of numerous drug candidates do not let them pass through the first phase of clinical trials. These challenges create a demand for alternative forms of drug delivery that surpass both biological and psychosocial barriers on the road to precision medicine.

The implantable drug delivery system (IDDS) is a medical device that can be surgically placed inside patient tissues to introduce a therapeutic substance and improve its efficacy and safety by controlling the rate, time, and place of drug release in the body [4]. IDDS represents a smart interface between the biological target and the drug depot. Importantly, for the regulatory purposes, IDDSs are combination products that merge two or more regulated components such as drugs, medical devices, or biological products that function as a single entity [5,6].

The following features are most commonly listed as the advantages of IDDS: (1) precise distribution of the drug to the target tissue without bioavailability and first-pass metabolism concerns that allows reduction in the active dosage; (2) minimization of side effects due to lower active substance systemic concentrations and absence of risk of incorrect drug administration; (3) prolonged and dose-controlled delivery of the drug, which makes the therapy independent of patient compliance [7,8,9]. Moreover, there are numerous possibilities for the smart IDDS equipped with sensors and feedback-controlled drug release IDDS, i.e., epileptic seizure preventing implants [10] or insulin pumps with glucose level analyzers [11].

It is considered that the first applications of IDDSs were subcutaneous implantations of hormone-containing pellets in cattle and poultry in the 1930s [8,12]. Bishop reported the first clinical use of IDDS for hormonal therapy in women [13,14]. Then, a few more implantable drug formulations were briefly studied [15]. Technologically, the era of the modern IDDS started in 1960s [9], when Folkman and Long demonstrated the application of a capsular (reservoir-type) device comprising a silicone rubber “Silastic” together with a semipermeable membrane, which used as a carrier for prolonged drug delivery to the ventricular myocardium in dogs with modeled heart block [16]. In this seminal thoughtful experimental study, the effects of six (mostly antiarrhythmic) drugs were recorded electrocardiographically. In addition, the gradient of the drug concentration forming in the vicinity of the implant was revealed via radiography detection of the thyroid I^125^ delivered to the myocardium by the “Silastic” IDDS. While the desired antiarrhythmic effects have not been achieved, this work revealed both a great potential of IDDSs for the targeted administration of therapeutics as well as a serious challenge related to the drug distribution from the implant to the tissues and the role of fibrotic reaction at the implantation site.

The clinical expansion of IDDSs was launched in the 1990s after the levonorgestrel-containing contraceptive implant Norplant^®^, based on Folkman’s and Long’s “Silastic” capsule schematics, obtained Food and Drug Administration (FDA) approval [15]. Currently, commercially available IDDSs are applied for the treatment of chronic diseases, pregnancy control and women’s health, pain management and mental health, and guided regeneration.

**Figure 1:**
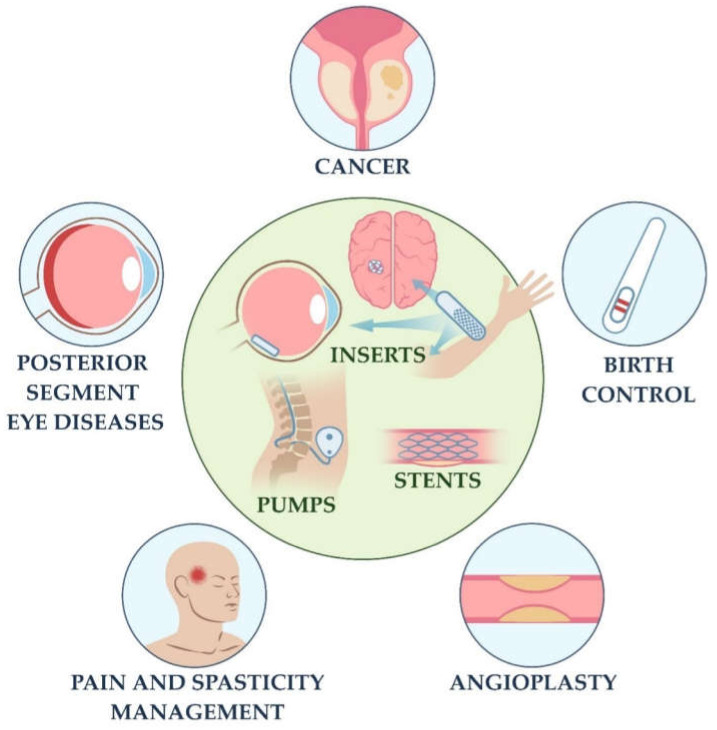
The main classes of commercially available IDDSs and the areas of their applications. Here, the term “inserts” includes solid implants introduced to the body via surgical manipulation. Insert IDDSs may release the drug by diffusion or by osmotic gradients (therefore, osmotic pumps can also be termed inserts). The term “pumps” mostly corresponds to the reservoir type of IDDSs that have a special mechanism for stimulating and controlling drug release. Note that some inserts employ osmotic gradient and can also be considered as pumps. Drug-eluting stents are placed inside the lumen of tubular anatomical structures (mostly blood vessels) to simultaneously preserve and restore the lumen and prevent excessive outgrowth of the neointimal tissue. Inserts are used for all listed clinical applications, excepting pain and spasticity management. Stents are applied mostly for angioplasty, but also for other types of lumen-supporting operations performed on tubular anatomical structures. Applications of pumps are diverse and defined by the type of the delivered drug. The most common application of pumps is in control of pain/spasticity and in cancer treatment. Image created with BioRender.com.

The IDDSs that reached the clinical and market stage can be broadly classified as inserts, pumps, and stents (Figure 1). Implants can be introduced into the body by injections or small incisions that require a short time and minimal anesthetic support (e.g., inserts, osmotic pumps for subcutaneous implantation) via intravascular interventions (stents) or by higher-volume operations (mechanical pumps). However, it has to be noted that there is also some fuzziness in the very terminology related to IDDSs. In particular, the terms “implant” and “insert” are often used interchangeably, sometimes merely for marketing reasons. In this review, we discuss solid implants that are “inserted” into the body by at least minimal surgical manipulation that involves the stabbing or incision of the tissues. According to the definition provided by FDA, an “implant” is a device that is placed into a surgically or naturally formed cavity of the human body that is intended to remain there for some time (usually at least 30 days, but shorter periods are also considered for safety reasons) [17].

The main therapeutic payloads that are delivered by an IDDS include hormonal, cytostatic, anticoagulant, antipsychotic, and metabolic drugs. There are also several emerging fields of IDDSs’ clinical use appeared at the advanced stages of clinical trials, e.g., mental health disorders.

While there is no established classification of IDDS, a few categories such as biodegradable and non-biodegradable devices, passive (inserts and stents) and dynamic (pumps) implants, electromechanical and reservoir-based systems, polymer-, or hydrogel-based ones, as well as location-specific implants such as ocular, subcutaneous, intracranial, etc., have been specified. This list, however, includes not only the clinically approved devices, but also those in various preclinical stages and in clinical trials. The type of the IDDS, in turn, defines the mechanism of the drug elution from the implanted system. (e.g., dominated by either passive diffusion, supported by artificial osmotic gradients, or enhanced by mechanically , thermally , magnetically , etc.–activated convection). The diversity of IDDSs, including the material, engineering, and drug release features, is comprehensively reviewed elsewhere [7,9,12,14,15,18,19,20]. For the purposes of this review, the most important categories are the anatomical placement of the IDDS, whether it has to be removed from the body after some time, and whether and to what extent it is biodegradable or bioerodible.

The FDA definition of implants implies that IDDS is expected to be in a long-time contact with the surrounding tissues and body liquids. According to the current safety standards, all implantable materials and devices must meet the criteria for biocompatibility to be considered for clinical approval [21]. The local immune response resulting from the interactions between the implant and the surrounding tissues is known as a foreign body reaction (FBR) [22,23].

**Figure 2:**
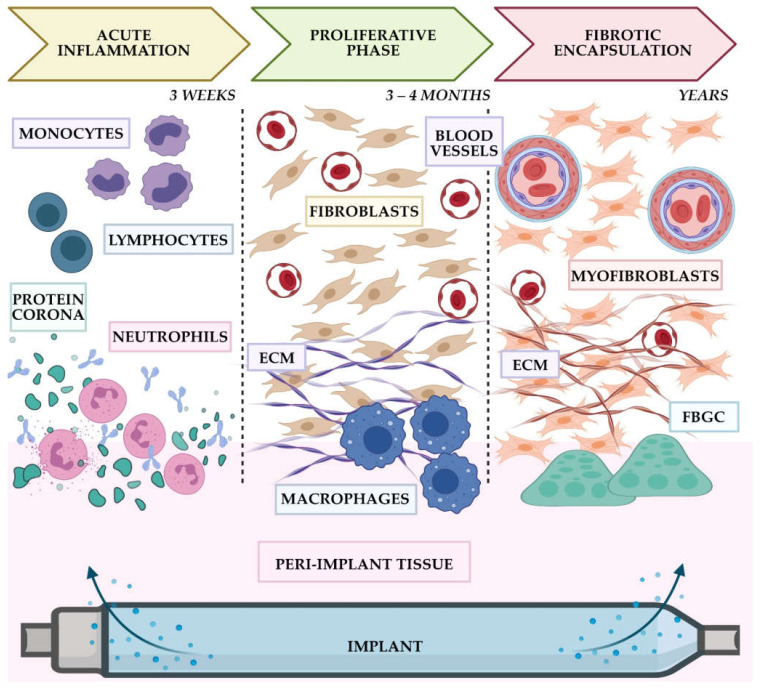
Schematic representation of the stages of foreign body reaction (FBR) to an IDDS (“implant”). The acute inflammation phase initiates the tissue response to the implantation and defines the composition of the provisional matrix and the cell adhesion efficiency. The proliferative phase is characterized by formation of granulation tissue. This tissue contains multiple blood capillaries that reinforce the soft provisional matrix. Then, the pericytes and endothelial cells of granulation tissue, as well as the bone marrow-derived progenitors, start to differentiate into fibroblasts. The cells of mature granulation tissue produce excessive amounts of extracellular matrix, including collagen fibers. Macrophages enhance this response. Further maturation of granulation tissue is associated with the emerging population of myofibroblasts. These cells not only secret matrix proteins but also contribute to the crosslinking of collagen fibers, their alignment, and resorption of the unloaded fibrillar elements, and contraction of the peri-implant fibrous connective tissue. Macrophages form foreign body giant cells (FBGC) surrounding the IDDS. Peri-implant vasculature also remodels, with reduction in the number of capillaries and increased presence of venules, arterioles, small caliber arteries, and veins. Note that the fibrotic peri-implant capsule may remain in the state of continuous remodeling for long time (months or years). Image created with BioRender.com.

FBR is a universal protective mechanism aimed at isolating the unrecognized poorly biodegradable object from the surrounding tissues and the body as a whole by the formation of a fibrotic capsule. The FBR develops as a chronic inflammatory process associated with the implantation of artificial objects such as IDDSs. It has three main stages: acute inflammation, proliferative phase, and fibrotic encapsulation of the implant [23,24] (Figure 2).

First, immediately after the surgical placement of the implant into the patient’s tissues, blood proteins absorb on the implant surface. This process is called protein corona formation [25]. As a result, all exposed surfaces of the IDDS become covered with the adsorbed proteins. Importantly, the composition and the stability of protein corona are strongly influenced by the bulk material and surface properties of the implants [25,26,27,28,29]. On the other hand, the absorption of proteins of the implant surfaces, especially the fibronectin, vitronectin, and fibrin, mediate cell adhesion and to a great extent determine the further course of FBR as well as the quality of the device–tissue integration [24,27].

The corona formation makes the implant “visible” to immune cells emerged in pro-inflammatory cytokine and chemokine secretion and direct cell–cell contact [25]. During the first few days after the implantation, the implant is surrounded by immune cell infiltrates that are predominantly composed of lymphocytes, macrophages, and mast cells. A provisional extracellular matrix (ECM) is formed on the base of blood-derived fibrin.

Next, the peri-implant granulation tissue formed by capillary loops and fibroblasts starts to differentiate from the bone marrow progenitors, blood vessel pericytes, and endothelial cells or migrate from surrounding tissues, resulting in the gradual maturation of the granulations and their transformation in more and more dense connective tissue. This stage is characterized by the active synthesis of ECM structural proteins, such as fibrillar collagens and associated glycoproteins. At the same time, macrophages locating in direct contact with the low- or non-biodegradable implant start to form foreign body giant cells (FBGCs), or giant cells.

Finally, the inflammatory-infiltrated granulation tissue and ECM formed during the proliferative phase gradually transform into a dense fibrous connective tissue that encircles the implanted materials. By skewing towards a profibrotic phenotype, FBGCs and macrophages facilitate the transdifferentiation of fibroblasts, pericytes, and endothelial cells into myofibroblast-overproducing collagen, causing IDDS fibrotic encapsulation and connective tissue capsule contraction. In the following months and years, the capsule becomes thinner but denser, which may affect the integrity of the implant.

While it is commonly assumed that the approved IDDSs should not induce a significant FBR (as they have passed the long way of pre-clinical and clinical testing), in fact, the immunogenicity of the long-lasting implantable and injectable products represents a recognized problem that may have significant impact on the treatment efficiency, safety, and outcomes [30]. However, the IDDS-associated FBR has not been analyzed systematically for multiple types of implants over time. Therefore, it is not clear whether the signs of FBR are present and associated with the clinically used IDDSs and to what extent these signs correspond to IDDS-related complications.

This review explores the landscape of all commercially available IDDS that have ever received FDA approval for clinical use. To the best of our knowledge, the information accumulated here is comprehensive and up to date upon completion of the review preparation in November 2021. In parallel, we analyze the available information on the IDDS-associated FBR manifestations, the biocompatibility of IDDS, and the role of biomaterial–host tissue interface in devices’ functionality. The exclusion criteria involved registered devices providing bolus drug release (e.g., infuse bone graft), as well as tablets, capsules, vaginal rings, intrauterine systems, skin patches, insulin pump inserts, vaccines, retard and depot forms of drugs, as well as various non-surgically placeable long-lasting drug delivery systems.

## 2. IDDSs and FBR: Current Clinical Landscape

### 2.1. Subcutaneous IDDSs

Subcutaneous IDDSs are currently used for many diverse clinical indications. Usually, these IDDSs are utilized for prolonged systemic drug delivery (commonly, implying the release of the drug over 3–12 months) in the absence of the patient control of the dosage. The FBR to these implants does not directly affect the target tissue due to its distant localization, while the inflammatory and fibrotic manifestations may take place in the vicinity of the implant. An overview of the FDA-approved subcutaneous IDDSs is presented in Table 1.

The implantable testosterone pellet, known as **Testopel**, was an early stage subcutaneous IDDS that received FDA approval in 1972 and was widely introduced to clinical practice for the treatment of testosterone deficiency syndrome. These pellets are biodegradable drug delivery implants that do not require surgical removal. **Testopel** is prescribed to patients with testosterone deficiency syndrome and consists of multiple drug-eluting polymer pellets that are implanted under the skin of the lateral abdominal wall or the lateral aspects of the buttocks [31]. **Testopel** supports therapeutic levels of testosterone for 4–6 months. However, it was shown that sometimes scars are present in the implantation site even after the pellets dissolve, and this forces physicians to choose a new implantation site for every implantation [32].

**Figure 3:**
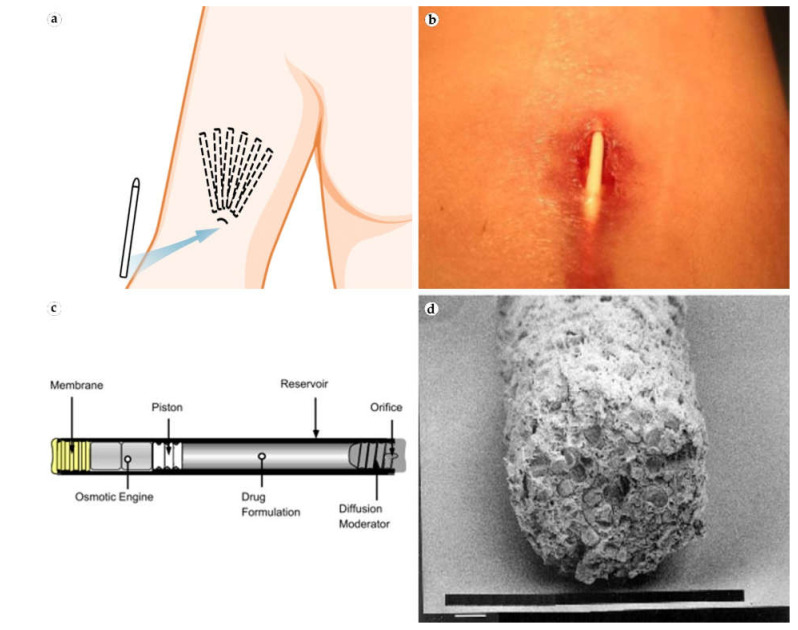
(**a**) **Norplant** rods’ subcutaneous positioning in the implantation area of the upper arm. Image created with BioRender.com. (**b**) Subdermal tissue reaction to **Nexplanon**. Note the wound breakdown and discharge with partial exposure of the implant above the skin surface. Reproduced from [33], with permission from BMJ Publishing Group Ltd. (**c**) DUROS osmotic minipump technology, which is a carrier for **Viadur** leuprolide-releasing implants. Reproduced from [34], with permission from Elsevier. (**d**) SEM of cross-section a single rod of Probufine implant showing a homogeneous mix of ethylene vinyl acetate and buprenorphine. Reproduced from [35], with permission from Oxford University Press and Copyright Clearance Center.

The levonorgestrel-releasing IDDS **Norplant** was the first modern-technology IDDS that received FDA approval, which was granted in 1990. The implant consisted of six drug-loaded silicone rods implanted subcutaneously in a fan manner under the skin of the shoulder medial surface (Figure 3a). The implant provided five years of effective pregnancy prevention. However, the application of **Norplant** was discontinued following the accumulation of critical reports on the complications associated with the device migration and the difficulties with the surgical removal of the IDDS due to excessive skin scarring.

In response to this critique, a two-rod version of the **Norplant**, the **Jadelle**, was proposed. It obtained FDA approval in 1996 and shows the therapeutic efficiency similar to **Norplant** with less frequent complications. This product was discontinued in the US, but it is still used in other countries [36]. For both implants there were multiple reports indicating serious adverse side effects, notably associated with the hormonal activity of the drug [37]. Several cases of implantation site complications, including infections at the insertion site and local atrophy of subcutaneous adipose tissue, were reported. However, neither peri-implant fibrosis nor other signs of FBR have been specifically mentioned [38,39].

**Implanon** is an FDA-approved single-rod implantable contraceptive containing 68 mg etonogestrel. It is widely used around the world and has earned the reputation of a safe and effective measure for pregnancy control. **Implanon** is implanted under the skin on the hand and provides sufficient daily release of etonogestrel for three to five years [40]. FBRs to Implanon were reported only in a couple of case reports [41,42]. There were no biopsy studies; however, a late-onset reaction in one case and antibiotic-resistant implant rejection causing protrusion through the skin surface in another patient pointed to the causative role of chronic inflammation. A common complication that both physicians and patients frequently reported was implant migration, usually in cranial direction [43].

For this reason, a modern model of an etonogestrel IDDS containing radiopaque ingredient barium sulfate, **Nexplanon**, was released to the market. An observational risk assessment study for **Nexplanon** collected information from physicians on 4373 **Nexplanon** removals. The study reported that the most common challenge was the encasement of the implant within fibrotic tissue (*N* = 29). Several physicians reported cases of patients with late-onset antibiotic-resistant Nexplanon implantation site inflammatory reactions (Figure 3b) [33,44]. However, one of the patients developed the inflammatory reaction after she had a beneficial experience with Implanon, which suggests a possible reaction to a new chemical composition, including barium. In another case, histologic examination showed active macrophagic and mastocytic reactions of the tissue surrounding Nexplanon [45].

Leuprolide-releasing implant **Viadur** was the first solid IDDS for patients with prostate cancer [46]. It is composed of a titanium cylinder with osmotically driven drug release that allows 12 months of maintaining of the therapeutic concentration (Figure 3c). The device was available in US between 2000 and 2007. However, the implant’s size was quite significant (4 mm × 45 mm), and clinical data on the market phase was very limited. Nevertheless, the delivery system was shown to be highly biocompatible [47].

**Vantas** and **Supprelin LA** are histrelin-releasing microporous polymer IDDSs. V**antas** is used to deliver histrelin in patients with advanced prostate cancer with daily dosages of 50 mg for 1 year [48]. **Supprelin LA** delivers 65 mg of histrelin daily in children with central precocious puberty for the same period [49]. Interestingly, both IDDSs are candidates for medical puberty retardation in youth with gender dysphoria [50]. There is only one reported case of possible FBR to histrelin-releasing **Supprelin LA** implant where sterile abscesses formed two times after the implantation of IDDS under the skin on different hands [51].

The most recent addition to FDA-approved subcutaneous IDDSs is **Probuphine**, a buprenorphine-releasing solid polymer implant for maintenance treatment of opioid use disorder. The implant system consists of four rods implanted under the skin of the hand, resembling the alignment of the **Norplant** elements [52]. **Probuphine** delivers active drug over a 6-month period, allowing patients to live a full social life without daily visits to physicians. No adverse FBRs were reported for this implant. However, in one clinical case, Probuphine rods were explanted from fibrotic surrounding tissues 7 years after the insertion. Scanning electron microscopy did not reveal changes in implant structure or invasion by fibrotic tissue (Figure 3d) [53].

### 2.2. Pump IDDSs

Another group of IDDSs are two-component implantable pumps for the intrathecal and intrahepatic drug delivery. Such devices consist of a subcutaneously implanted drug-containing metallic reservoir and a flexible silicone catheter placed subcutaneously and connecting the reservoir with the intrathecal space in order to deliver the drug to the cerebrospinal fluid or to the bloodstream via blood vessels supplying the target organ. An overview of the FDA-approved pump IDDSs for intrathecal and targeted intraorgan drug delivery is shown in Table 2.

The **Infusaid pump** was the first implantable intrathecal drug delivery system that received FDA approval, which was given in 1982. The introduction of the **Infusaid** rapidly popularized the concept of the direct delivery of the drug to the target tissue and inspired further applications of this pump in various fields of medicine. Originally, this IDDS was approved for the delivery of heparin and floxuridine to treat the liver-located malignancies. However, the system was soon made commercially available and employed for the delivery of insulin, morphine, 5-Fluorourocil, methotrexate, glycerin, bleomycin, cisplatin, amikacin, and netilmicin [54]. However, the application of **Infusaid** for the delivery of chemotherapy drugs through the hepatic artery led to serious hepatobiliary toxicity in most patients [55]. Moreover, severe fibrosis of the extrahepatic biliary system was reported as a complication of hepatic artery infusion of floxuridine [56]. **Infusaid** application as an artificial pancreas for patients with diabetes was accompanied by a frequent rate of severe complications, including obturations of the intraabdominal catheter [57]. All these reasons, together with increasing numbers of reports on the spinal cord damage caused by intrathecal catheters, resulted in the discontinuation of this pump.

**Figure 4:**
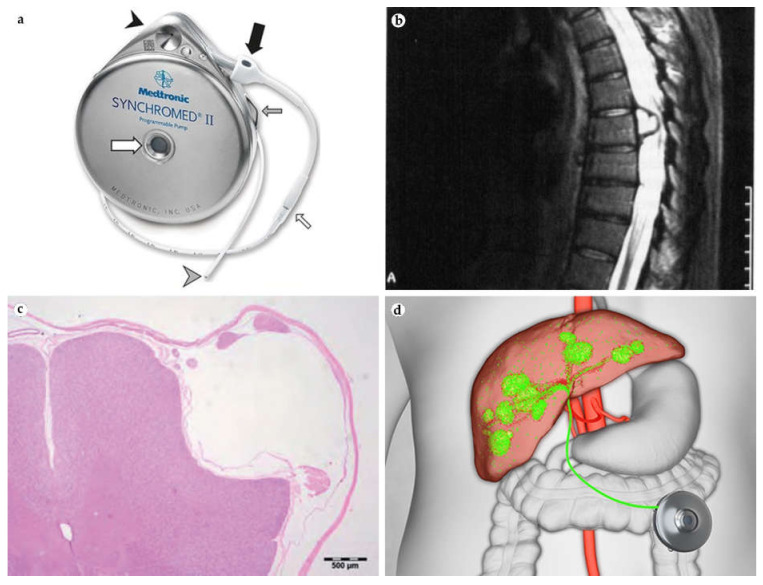
(**a**) External view of implanted **Synchromed II** pump and intrathecal catheter 8731SC. Pump with the catheter access port (black arrowhead), pump catheter connection (thick black arrow), refill membrane (thick white arrow) and suture loops for fixation (thin grey arrow), catheter–catheter segment connection (thin white arrow), and titanium catheter end (grey arrowhead). Reproduced from [69] under the terms of the Creative Commons CC BY license. (**b**) Gadolinium-enhanced T1 weighted sagittal MRI image showing extrinsic mass formed in the area of catheter insertion and compressing the spinal cord at T7 after implantation of **Synchromed** pump with permanent catheter (#8703) for the delivery of analgesic drugs. After 4.5 years of intrathecal drug delivery, the patient developed increasing neurological deficit and paresis. The pathology examination revealed a cyst containing sterile eosinophilic liquid material without leucocytes. The cyst capsule was infiltrated with lymphocytes. Reproduced from [34], with permission from WILEY. (**c**) Spinal cord compression caused by the catheter of a non-identified intrathecal pump IDDS without morphine elution. Note the evident compression of the spinal cord in the vicinity of the catheter track and absence of morphine-associated granuloma formation. Reproduced from [64], with permission from Oxford University Press and Copyright Clearance Center. (**d**) Scheme of hepatic artery infusion with **Intera 3000**. Reproduced with permission by Intera Oncology [70].

Later, **Infusaid** was replaced by the second generation of this IDDS [58]. **SynchroMed II** is the most widely used implantable pump for prolonged drug delivery (Figure 4a). Its original iteration, **SynchroMed**, was approved by FDA in 1991. The list of **Synchromed II** therapeutic payloads includes baclofen (for the reduction in severe spasticity), morphine, and Ziconotide (for pain management) [59,60].

Additionally, the FDA approved **Synchromed II** for the intravenous (through the vena cava superior) delivery of Treprostinil for patients with pulmonary arterial hypertension. The system consists of two separately inserted components—a reservoir (20 or 40 mL in volume) and a catheter. The reservoir is noticeably large since it is supplied with a battery providing up to 7 years of life. Catheters differ in length and form because of different routes of implantation. The major advantage of this pump system is the availability of the programming options, providing both physician and patient with a tool for fine and dynamic correction of the drug dosage.

Pump-associated complications can be divided into catheter- (migration, dislodgement, breakage, kinking, obstruction), pump- (rotation, malfunction, abnormal infusion), and surgery- (infection, cerebrospinal fluid leakage) related problems [61]. Soon after **SynchroMed** became commercially available, intrathecal catheter-associated complications were noted. In particular, aseptic granuloma formation with subsequent neurologic sequelae was observed [62]. In a survey among the neurosurgeons who implanted **SynchroMed** and **Infusaid** pumps, 31 (6%) of responders reported that their patients developed lesions around the implanted catheters (Figure 4b,c) [34]. Fifteen (43%) neurosurgeons reported paresis or paralysis, which remained irreversible in 10 patients. Most of the reported cases included histopathology examination data with microphotographs picturing granulomas. Later, these complications were reproduced in a sheep model demonstrating mild-to-moderate spinal cord compression 43 days after the operation [63]. Finally, a study to assess granulomatous responses to intrathecal catheters in canine model with multiple control groups (including **SynchroMed II pump**) revealed that granulomatous lesions development and spinal cord immune cell infiltrations were correlated with the dosage and concentration of morphine [64]. Saline-releasing catheters caused compensated spinal cord compression without fiber degeneration, while morphine catheters induced tissue infiltration with neutrophils, macrophages, and plasma cells. Scar transformation of the skin pocket occurs due to direct contact of the implant with aponeurosis and subcutaneous fat and repetitive refill injections [65]. As a result, two serious complications can happen—catheter disconnection and the prevention of accurate drug refilling with subsequent overdose [66,67].

**Intera 3000** (or **Codman 3000**) presented a novel design model, an infusion pump that did not require a battery as it operated via a gas–liquid propellent system driven by body temperature (Figure 4d). This innovation removed the need to replace the pump. The device was approved by the FDA for the intrathecal delivery of morphine and baclofen for pain management and hepatic arterial infusions for tumor site [68]. Despite promising results, the device was discontinued in 2018, which can be explained by high pricing and low demand (300 sales a year in US) [7]. Recently, the technology was acquired by Intera Oncology and currently, it is marketed as the only FDA-approved device for hepatic artery infusion of floxuridine. According to the data provided by Intera Oncology (in private communication), both the efficacy and safety of hepatic artery infusion via Intera 3000 pump when used with Floxuridine show notable improvement, as physicians have optimized the treatment protocols of patient care and toxicity monitoring.

The **Prometra II** intrathecal drug delivery system combines the best features of osmotic and infusion pumps through the addition of a precise battery-dependent valve delivery system. It entered the market as a morphine-delivering system and was approved by the FDA for baclofen treatment of spasticity across numerous conditions, including multiple sclerosis, in 2012 [7]. Because of the novelty of this system, there is no literature evidence concerning device-related complications yet.

### 2.3. Ocular IDDSs

**Figure 5:**
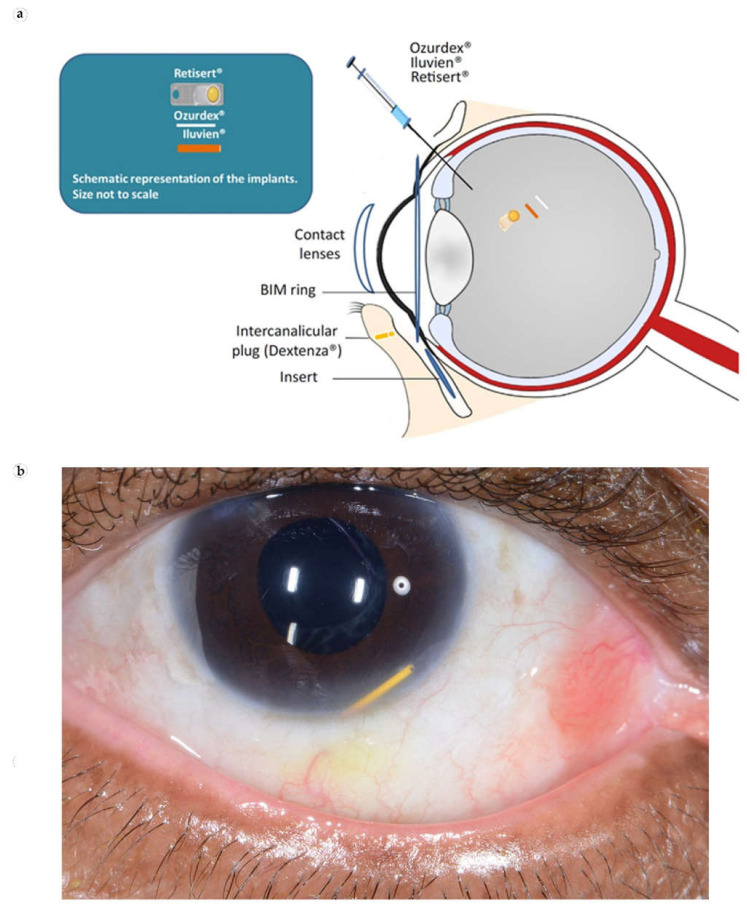
Ocular IDDSs. (**a**) Cross section of a human eye showing the inserting/injection positions of commercially available ocular IDDSs. Reproduced from [73], with permission from Elsevier. (**b**) Migration of **Iluvien** implant in the inferior angle of anterior chamber. Reproduced from [74], under the terms of the Creative Commons CC BY license.

The principal causes of irreversible blindness and visual impairment are retinal degenerative diseases, which affect millions of people around the world. It is estimated that about 9.1 million American adults have one of the major retinal degenerations, such as diabetic retinopathy, glaucoma, and macular degeneration [71]. The central route of local ophthalmic drug delivery remains the topical application of solutions to the surface of the eye in the form of drops. Local administration to the eye is effective in treating the surface of the eye and diseases of the anterior part of the eye such as conjunctivitis, blepharitis, keratitis, and dry eyes; it is of no value for posterior eye diseases. It has been estimated that typically less than 5% of a topically applied drug actually permeates the cornea and reaches intraocular tissues [72]. An overview of the ocular IDDSs is given in Table 3. The placement of commercially available ocular IDDS in the eye is shown in Figure 5a.

Currently, the treatment of diseases that affect the posterior eye segment is limited by the difficulty of administering effective doses of drugs to target tissues. Local administration strategies using intravitreal injection are effective in overcoming barriers associated with local and systemic administration of ophthalmic medications. Intraocular injections were the first effective back-of-the-eye therapy, most notably with the approval of ranibizumab (Lucentis, Genentech, Inc., San Francisco, California, USA) and the subsequent approval of aflibercept (EYLEA, Regeneron Pharmaceuticals) for treatment of wet age-related macular degeneration (AMD) [75]. Intravitreal injection is invasive and associated with serious side effects: bleeding, foreign body sensation, persistent discomfort, retinal detachment, cataract formation, and bacterial endophthalmitis [76]. Poor patient compliance, difficulty in administering drugs accurately, and variable drug efficacy can impede the achievement of desired therapeutic results in ocular diseases.

Intraocular implants, which are usually classified as non-biodegradable and biodegradable IDDS devices, are transplanted directly into the vitreous body. The installation of the IDDS device is invasive and can cause complications similar to those caused by intravitreal injections [72].

Typically, non-biodegradable IDDS devices entrap a drug within a reservoir surrounded by non-biodegradable release membranes [77]. They are usually composed of polyvinyl alcohol (PVA), ethylene vinyl acetate (EVA), and polysulfone capillary fiber (PCF) [78].

In 1996, the non-biodegradable implant **Vitrasert** was approved by the FDA for the treatment of cytomegalovirus (CMV) retinitis [79]. The implant is made using EVA and polyvinyl alcohol, which are coated with layers of granular ganciclovir. This device is introduced via pars plana incision and sutured to the sclera. A small hole in the EVA membrane provides controlled drug release through passive diffusion for 6–8 months [78]. The implant requires surgical removal. Postoperative complications occurred in 12% of the ganciclovir implant procedures and were associated with hematogenous retinal detachment, vitreous hemorrhage, endophthalmitis, and cystoid macular edema with epiretinal membrane [80]. The **Vitrasert** implant was withdrawn from the European market for human use in April 2002 by the European Medicines Evaluation Agency due to ocular complications (retinal detachment) [81].

The **Retisert** implant is used for the treatment of chronic non-infectious posterior uveitis. The intravitreal implant received FDA approval and became widely commercially available in 2005. **Retisert** is composed of a central core consisting of fluocinolone acetonide (FA) compressed into a 1.5 mm diameter pellet coated with a nonpermeable silicone capsule featuring an orifice to allow drug release. This device is implanted into the vitreous humor. It is also attached to a PVA suture tab and coated with extra PVA and silicon layers with a drug diffusion port, which delivers the medicament over ≤2.5 years but must then be removed [78]. **Retisert** implants tend to dissociate, leading to intraoperative complications, including posterior retinal tear and limited suprachoroidal hemorrhage [82]. The most frequently reported ocular adverse events in clinical trials with Retisert occurring in 50–90% of patients included cataract, increased intraocular pressure, procedural complications, and eye pain [83]. Based on clinical trials with this device, it was estimated that within 3 years post-implantation, approximately 77% of patients would require intraocular pressure (IOP)-lowering medications and 37% of patients would require filtering procedures to control IOP [84]. Additionally, there have been several cases of cytomegalovirus corneal endotheliitis following the implantation of fluocinolone releasing device for uveitis [85,86].

**Iluvien** implant (Figure 5b) is a recently FDA-approved, non-biodegradable DDS device providing a sustained release of fluocinolone acetonide formulation for the treatment of diabetic macular edema (DME). This device consists of a small cylindrical polyimide tube loaded with fluocinolone acetonide, which is released through membrane caps on both ends of the tube [87]. The implant is injected into the back of the eye using a 25 G needle, creating a self-healing hole. This device is designed to last up to 3 years, thereby minimizing systemic toxic effects. After 36 months, a new implant can be inserted without removing the previous implant, as no side effects have been reported from having multiple implants in the eye [88,89,90]. Ocular hypertension is one of the most common adverse events associated with the use of intraocular steroids [91]. Moreover, complicated cataract surgery and vitrectomy are possibly associated with the migration of the Iluvien implant into the anterior chamber [92].

Later, a very similar IDDS, **YUTIQ**, was granted FDA approval to treat chronic noninfectious uveitis affecting the posterior segment of the eye. It contained 0.18 mg of fluocinolone acetonide (compared to 0.19 mg) but provided the same release profile for 3 years [93].

The **Ozurdex** dexamethasone drug delivery system (Figure 5d) is a biodegradable intravitreal implant, which releases a small amount (700 µg) of glucocorticoid dexamethasone over a period of up to 6 months. It was approved by the FDA in 2009 as a first-line therapy for the treatment of macular edema following branch or central retinal vein occlusion, as well as for noninfectious posterior uveitis [94]. **Ozurdex** is injected into the vitreous cavity via a 22 G needle. The implant has a biphasic release of the drug with a loading dose for the initial 2 months, followed by a maintenance dose up to 6 months [95]. Sustained release is provided by the cylindrical poly (lactic-co-glycolic acid) (PLGA) matrix that dissolves completely in vivo. The main consequences of the intravitreal implantation of dexamethasone (**Ozurdex**) are ocular hypertension and cataracts [96]. Clinical studies have shown that ocular hypertension was recorded for 28.5% of injected eyes, and conjunctival hemorrhage (22%), eye pain (8%), conjunctival hyperemia (7%), cataract (5%), vitreous detachment (2%), headache (4%), and intraocular pressure-lowering medication was recorded for 31% of eyes [97,98]. Epiretinal fibrosis around the implant was reported as a postoperative complication resulting in a recurrent retinal detachment, the desegmentation of **Ozurdex** implant in the vitreous cavity, and the migration of implant into the anterior chamber [99,100,101,102].

**Dextenza** (a hydrogel intracanalicular plug) is approved for the treatment of pain and inflammation following cataract surgery, during which 0.4 mg of sustained release dexamethasone insert is placed inside the tubule to ensure consistent and uniform drug delivery to the ocular surface for 30 days after a single dose [103]. The dexamethasone insert is inserted through the punctum into the canaliculus, swells on hydration, anchors into place, and does not require surgical removal; it dissolves and leaves the nasolacrimal system after the treatment [104]. The most common ocular adverse reactions are: anterior chamber inflammation, including iritis and iridocyclitis (10%); increased intraocular pressure (6%); reduced visual acuity (2%); eye pain (1%); cystoid macular edema (1%); corneal edema (1%); and conjunctival hyperemia (1%).

Recently, the FDA approved several new ocular IDDSs. These include **Susvimo**, a surgically fixed permanent refillable implant, for the continuous release of the ranibizumab, a monoclonal antibody against vascular endothelial growth factor (anti-VEGF) to treat neovascular AMD [105]. The device provides 6 months of sustained drug release, replacing monthly intraocular injections [106]. Interestingly, it is the first IDDS to deliver monoclonal antibodies. Another example is the **BIM ring**, a Brimonidine intravitreal biodegradable implant for the treatment of dry AMD and retinitis pigmentosa with six months release [107]. Due to the short time after FDA approval, information on the FBR to these implants is not available yet.

Advances in biomaterials and nanotechnology have led to major growth in the research of ocular implants. For example, **I-vation**, a non-biodegradable polymeric implant, was intended to release triamcinolone for at least two years in patients with diabetic macular edema. This is helix-shaped, non-biodegradable implant was coated with triamcinolone. It was designed to release the drug over a period of 36 months in patients with diabetic macular edema. However, the device caused complications including increased intraocular pressure and cataracts and was terminated after phase two of clinical trials [108].

A different approach for prolonged drug delivery was realized with **Verisome** technology, a fully biodegradable liquid form of an active substance in the aqueous sphere. **Verisome**-based, dexamethasone-containing **DEXYCU** obtained FDA approval to treat postoperative inflammation in 2018 [109]. However, this specific form of drug delivery is closer to retard formulations than implants, and the technology has the potential to maintain the efficient drug concentration for months [110]. **Verisome** (IBI-20089) can be applied to deliver various types of drugs, including proteins, up to one year [111].

An encapsulated cell technology implant, **Renexus**, is undergoing clinical trials for the treatment of macular telangiectasia and glaucoma. Genetically modified cells deliver human ciliary neurotrophic factor (CNTF) over an 18-month period [112]. Phase three of clinical trials for the safety and efficacy of Renexus in macular telangiectasia (ClinicalTrials.gov Identifier: NCT03316300) will be completed in 2022. Moreover, phase two clinical trials of the dual intravitreal implantation of Renexus for the treatment of glaucoma is estimated to be completed by the end of 2023.

Finally, the recent developments in **drug-eluting contact lenses** reveal them as beneficial alternatives to anti-glaucoma drops and gels [113]. Consistency of low levels of the intraocular pressure is essential for the prevention of progressive loss of vision. Phase two of early glaucoma trial of LLT-BMT1 drug-eluting contact lens (ClinicalTrials.gov Identifier: NCT04577300) was completed earlier in 2021.

### 2.4. Neurological IDDS

Drug delivery to the brain is limited primarily due to the highly selective permeability of the blood–brain barrier. IDDSs are promising devices allowing drugs to bypass this limitation. Currently, the primary field of IDDSs intracerebral applications is the treatment of advanced malignant glial tumors.

There are only four drugs (temozolomide, lomustine, intravenous carmustine, bevacizumab) and one IDDSs (carmustine-eluting inserts, or “wafers”, produced under a commercial name **“Gliadel”**) that have been approved by FDA to treat malignant gliomas [114].

**Figure 6:**
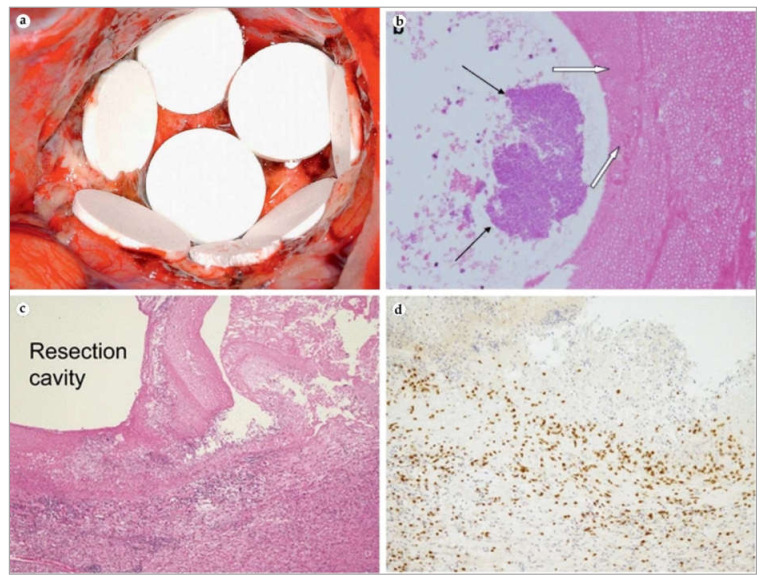
(**a**) **Gliadel** wafers in resection cavity. Reprinted from [117], with permission from Elsevier. (**b**) **Gliadel** wafer (white arrows)-associated bacterial growth (black arrows). Adapted from [118], with permission from Springer Nature. (**c**) Resection cavity where **Gliadel** was placed is surrounded by tissue infiltrated with immune cells. (**d**) Same clinical case: peri-implant tissue is filled with numerous CD^8+^ T-cells. (**c**,**d**) Adapted from [119], with permission from Springer Nature.

**Gliadel** is a polymeric biodegradable carmustine-containing, disc-shaped implant (Figure 6a). This IDDS was approved for the treatment of recurrent high-grade gliomas in 1996. In 2003, the approval was extended to include patients with primary glioblastomas with planned surgical resection. Eight wafers are directly implanted into tumor resection cavity for site-specific chemotherapy. A decade-long multi-institutional studies revealed that **Gliadel** prolongs overall survival to 14–20 months compared to 12 months for patients only treated with radiotherapy [115,116]. Large retrospective analysis of a thousand craniotomies indicted that there was no significant difference in complication rates between **Gliadel** and simple resection groups, in particular in perioperative surgical site infection (Figure 6b), inflammation (Figure 6c,d), cerebrospinal fluid leak, meningitis, wound healing difficulty, symptomatic malignant edema, 3-month seizure incidence, deep vein thrombosis, or pulmonary embolism [116].

Nontumorous brain was shown to react to **Gliadel** wafers. A prospective study evaluated the phenotypes of immune cells surrounding the implants and clearly demonstrated that the numbers of CD^8+^ and CD^68+^ cells were significantly higher in this tissue than in control resected tissues without the IDDS. The authors connected the results to the antitumorous effect of the wafer since the recruitment of cancer-killing CD^8+^ T lymphocytes (Figure 6d) are inflammation-regulating CD68^+^ macrophages that can support treatment with carmustine [119]. Later, the same group published an article on long-term follow-up after **Gliadel** implantation with presented histological findings on macrophage patterns. The initial tumor site had no evident tumor cells while having multiple CD68^+^ positive cells. This proportion was reversed in distal tissue locations [120]. Over the years, case reports connected **Gliadel** wafers with bacterial infection and local vessel reactions [118,121]. However, the understanding of relatively short-term host tissue reaction to brain implants is important for the design of near future intracranial implants against a range of neurological disorders. **Gliadel** wafers are still commercially available; however, they have not become the standard of care and slowly lost the interest of the neurosurgical community due to significant variabilities in overall survival rates between institutions and high costs.

The neurological implant research field is not limited to brain IDDS. The drug delivering nerve guidance conduits are expected to emerge soon from the clinical trials [122]. Additionally, there is a number of clinical trials focusing on cell and drug delivery for spinal cord injury, aiming at controlling the inflammation [123].

### 2.5. Cardiovascular IDDSs

Balloon coronary angioplasty is a standard of care for patients with narrowed coronary or peripheral arteries. Mechanical reperfusion with stent implantation is a life-saving operation for patients with acute infarction. The first implants, bare metal stents, were highly effective but caused in-stent restenosis after 6–12 months [15]. This complication is defined by the organization of fibrin surrounding the stent followed by neointimal proliferation. Ingrowing smooth muscle cells and myofibroblasts cause the overproduction of collagen fibers until this process stabilizes with endothelialization. Importantly, the neointima can accumulate calcium and lipid deposits, making the blood vessel dangerously unstable.

**Figure 7:**
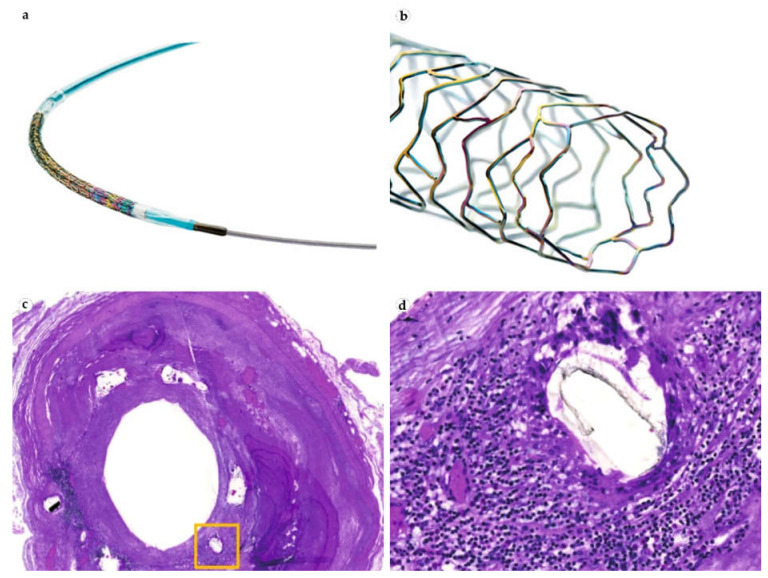
**Vascular drug**-**eluting stents** (**DES**)**.** (**a**) The undeployed Orsiro DES on its delivery system. (**b**) Close-up view of **Orsiro’s** ultrathin struts in their expanded state. (**a**,**b**) Images courtesy of BIOTRONIK AG, Bülach, Switzerland. (**c**) Histological image of **Cypher** Sirolimus-eluting stent implanted over a fibrocalcific plaque. (**d**) Same clinical case: severe inflammatory reaction localized near the stent segments. (**c**,**d**) Reprinted from [127], with permission from Springer Nature.

The drug-eluting stents allow control of the blood vessels’ intima proliferation layer and foreign body-mediated thrombosis by using a special class of immunosuppressant/antiproliferative/anti-migrating drugs, the mTOR inhibitors [124]. First-generation vascular IDDS (**Cypher**, **Taxus**, **Xience**, **Promus**, **Endeavor**, **Resolute**) were coated with permanent polymers delivering antiproliferative drugs over a period of 90–180 days(paclitaxel (cytostatic), sirolimus, everolimus and zotarolimus (mTOR inhibitors)) (Table 4 and Figure 7). Restenosis rates after the implantations of such IDDSs dropped significantly in comparison to bare metal stents. However, the observed risk of thrombosis and around 10% of restenosis prevalence charted the direction for subsequent modifications.

The second generation of vascular IDDS (**Orsiro**, **Synergy**) were based on the bioresorbable polymer coatings of metal stents and delivered sirolimus, everolimus, and biolimus for 30–180 days. Sadly, the prevalence of restenosis was still 5–10%, which could be explained by the effects of both antiproliferative drugs and products of polymer degradation. The walls of the reperfused vessels were morphologically studied and showed delayed intimal proliferation and focused on chronic inflammatory reaction. Another complication of these stents was the development of neoatherosclerosis [125].

The most recent direction of stent engineering explores the possibility of a fully resorbable polymer stent. The FDA approved the first fully absorbable stent, **Absorb GT1 Bioresorbable Vascular Scaffold System**, to treat coronary artery disease in 2016. While there are no serious concerns about the immediate biointegration and inhibition of thrombosis of these constructs, there is an ongoing discourse about the risks of the stent fracture and subsequent blood vessel rupture due to the progressive loss of mechanical stiffness over the period of resorption.

Cardiac patches, biomaterial carriers, are expected to become future standard-of-care devices. Their ability to deliver cells and growth factors to facilitate heart tissue regeneration was shown in animal models. Moreover, there is a great possibility of developing on-demand, drug-releasing systems, which can be extremely useful for patients with heart diseases [126].

## 3. General Discussion and Future Prospects

### 3.1. Main Findings

The current clinical landscape of clinically (FDA) approved IDDSs includes subcutaneous, ocular, and intracerebral solid implants/inserts, as well as various pumps and drug-eluting stents (see Figure 1). The summary information about the IDDSs discussed in the current review is presented in Table 5. As can be seen from this table, to date, the majority of the commercially available IDDSs belong to non-biodegradable implants. Most of subcutaneous IDDSs and all infusion pumps require surgical removal at the end of the designated period. In contrast, ocular IDDSs, intracerebral inserts, and drug-eluting stents can usually be left in the tissues without removal. Reservoir implants predominate among subcutaneous and ocular IDDSs, while the drug-eluting matrices are used in stents and, much less often, in subcutaneous, ocular, and intracerebral inserts.

The non-biodegradable nature of the majority of the currently commercially available IDDSs implies inevitable FBRs. However, the direct pieces of evidence indicating the fibrotic complications and excessive tissue growth at the implantation sites are relatively commonly reported only for subcutaneous inserts and for infusion pumps. At the same time, these types of adverse reactions are uncommon for ocular implants and drug-eluting stents. The complications linked to infections and the local effects of the eluted drugs are mostly non-specific to the IDDS itself. Additionally, the information available so far does not allow “blaming” of any specific vehicle material applied in IDDSs’ construction in the observed adverse responses (Table 6).

Key factors of FBR to the clinically used IDDS. The analysis of the data presented in this review allowed us to nominate a leading triad of the factors that significantly contribute to the FBR-related complications associated with the use of current IDDSs. Our analysis shows that this triad includes:

(1) Implantation site (the immune reactivity of the host tissue);
(2) The volume of the implant (defining the volume of surgical trauma and the local tissue extension);
(3) The drug load of IDDS (anti-inflammatory, anti-proliferative, and immunosuppressing drugs contribute to less prominent FBR).

One of the most unexpected findings emerging from the analysis of the current clinical landscape of IDDSs is that ***the engineering aspects of the modern IDDSs are addressed much better than the biology of FBR***. It seems that there is a very good understanding of the efficient ways to create good implantable devices for drug delivery, and these principles can be re-used in new IDDSs with high chances of success. However, the tissue response such as *** FBR still remains a challenging target***. It certainly can be controlled further. This requires more research but also allows more room for improvement.

### 3.2. Analysis of the Application-Specific Trends

We currently view IDDSs not as a separate medical technology but rather as a strong bioengineering trend with emerging applications in diverse clinical fields. However, this trend meets many sides of a universal host tissue response, the FBR. The analysis of the complications associated with different kinds of implantable systems indicates that many of the current challenges have non-specific solutions that will be implemented in systems developed in the near future.

The history of the subcutaneous implantations of IDDSs in the upper-arm area proves that the minimization of the quantity of the system components and their sizes can be efficient for the long-term satisfactory patient experience. Moreover, the transition from Norplant, with six silicone rods, to Implanon, with one EVA rod, was better met by both patients and physicians because of the reduced volume of surgical procedures. Reports about inflammatory complications associated with subcutaneous implants are now scarce and indicate rare individual adverse FBR responses. The most discussed complication of upper-arm IDDS implantations is their possible migration towards the forearm area, which must be regularly monitored by the patient. The addition of the imaging tracer compound has not resulted in significant progress due to the problem of the device’s under-skin mobility. The current major trend in this field is the development of one-rod systems with reliable fixation of the implant in the tissue and predictable pharmacokinetics of the drug. This challenge, possibly, has two general solutions: (1) to create constant semi-permeable skin pockets for implants, or (2) to modulate FBR to control the implant’s position and functionality. From the observations of IDDSs used for intrathecal drug delivery (where the reservoir parts are placed in the skin pocket), it seems that the first approach can result in the increased incidence of missed injections and cosmetic defects.

Implantable pumps’ applications reveal another, partially surprising, observation. First of all, only a small number of these IDDSs obtained FDA approval. The initial enthusiasm and expectations that any drug can become more efficient if it is delivered locally by a well-controlled mechanical pump soon met with the disappointment of the medical community following a massive promotion of a relatively raw technology. It took 25 years after the Infusaid pump became commercially available to reveal the source of the catheter-associated neurological sequelae. Implantable pumps did not become the standard of care for chemotherapy or diabetes treatment, but they found their niche in pain and spasticity management, where intrathecal delivery is crucial. Currently, we observe very careful and safe evolution of this technology in extension of application areas, the miniaturization of pump sizes and procedure protocols for drug refills, and doctor–patient dynamic dosage control.

Intraocular implant technology has changed dramatically over the last 15 years. Earlier models demanded surgical fixation and removal, causing complications including fibrotic encapsulation. The most recent approach to make the IDDS so small that they can be delivered with a syringe paid off because of the closed space of vitreous body and immune privilege of the eye. This allows ophthalmologists to implant several IDDS; however, there is a need to control their possible migration to the anterior chamber of the eye.

The only FDA-approved intracranial IDDS is Gliadel wafers. While there was no significant alteration of the technology, the adaptation of procedure protocols allowed the prolonged overall survival of patients with high-grade gliomas from 2 to 10 more months of life. This case clearly presents that IDDS technology is not about the search for panacea but about gaining maximum results from the medicines we have at hand.

Cardiovascular surgery has the richest selection of IDDS. Unlike all implants mentioned above, drug-eluting stents were specifically designed for better biointegration with the blood vessel walls and serum proteins. These stents do not cancel the proliferation of neointima or stop angiogenesis but slow this process through the specific targeting of the mTOR pathway, which allows for the preservation of the required diameter of the lumen. In the next several years, we expect to observe how fully bioresorbable stents will take their place in the field. It is important, however, to continue monitoring possible defects and subsequent hemorrhages that can occur to semi-resorbed stents from mechanical stress. In addition, lessons learnt from the application of cardiovascular IDDSs point out the positive role of combining the materials with different properties, the usage of advanced polymer compositions, and mild and precise suppression of the FBR at the proliferation stage.

Finally, it is important to remember that the IDDS–tissue interactions have two contributors. With the enormous potential of bioengineering to control the implant side, it is also very important to consider the local tissue contexts of the implantation site. The well-vascularized and immune active organs, such as skin and brain, may require more precise control of FBR, including the initial stages of acute inflammation to ensure the required level of cells’ adhesion to the device and its eventual integration. The vascular niche of the drug-eluting stents as well as the vitreous matter surrounding the ocular IDDSs are intrinsically inflammatory due to the disease that required treatment, while, certainly, they provide less extensive exposure to certain cell populations. Possibly, other drugs able to control local tissue proliferation at the implantation site (for various types of IDDSs and their anatomical locations) may enter the scene. Our own recent studies have demonstrated a possibility of re-purposing pirfenidone for FBR-controlling IDDS [129]. Taken together, these trends may indicate a future direction of exploration and whether these features can be reconstructed for the implants placed in other tissues.

## 4. Conclusions

IDDSs represent a very attractive therapeutic strategy that bypasses the limitations of the conventional drug administration routes. This technology contributes to the introduction of new bioactive substances to the clinical practice and helps to repurpose established medicines. It brings together physicians, researchers, engineers, industry companies, and venture business communities that have the energy and intentions to improve the quality of the patients’ lives and enhance the efficiency of treatment outcomes. The avalanche of clinical trials for the new IDDSs is anticipated to occur in the near future. However, we encourage clinicians and researchers to pay their attention to the lessons pointing at the importance of implant–tissue biointegration, which became clear during the formation of the current clinical landscape of IDDS technology. Most importantly, the experience of clinical application of IDDSs indicates the need to concentrate the efforts on the control of FBR via the biological mechanisms rather than by further increasing of the complexity of the engineering solutions. We believe that the current review has thrown some light on this important problem.

## Figures and Tables

**Table 1:** FDA-approved subcutaneous IDDSs.

IDDS	FDA Approval Date	Drug ^1^	Drug Class ^2^	Drug Release Duration	Material ^3^	Indication
Testopel	1972	TS	Hormone	4–6 months	PVP	Testosterone deficiency syndrome
Norplant (Jadelle)	1990	LG	PG	5 years	Silicone, PDMS	Pregnancy control
Implanon (Nexplanon)	2006	EG	PG	3–5 years	EVA	Pregnancy control
Viadur	200	LP	GTRHA	1 year	Titanium	Prostate cancer
Vantas/ Supprelin LA	2004/2007	HS	GTRHA	1 year	EVA	Advanced prostate cancer/central precocious puberty
Probuphine	2016	BPH	Opioid	6 months	EVA	Opioid use disorder

Abbreviations: ^1^ LG, Levonorgestrel, EG, Etonogestrel, TS, Testosterone, LP, Leuprolide, HS, Histrelin, BPH, Buprenorphine; ^2^ PG, Progestogen, GTRHA, Gonadotropin-releasing hormone analogue; ^3^ PVP, Polyvinylpyrrolidone; PDMS, Polydimethylsiloxane; EVA, Ethylene Vinyl Acetate.

**Table 2:** FDA-approved IDDSs for intrathecal drug delivery.

Implant	FDA Approval Date	Drug	Indication
Infusaid pump	1982	Heparin, floxuridine, fluorouracil, amikacin	Recurrent thromboembolic disease, hepatic arterial infusions for tumor site, osteomyelitis
SynchroMed (SynchroMed II)	1991	Baclofen, Morphine, Ziconotide, Treprostinil	Severe spasticity, pain management, pulmonary arterial hypertension
Intera 3000 (Codman 3000)	2011 (1996)	Morphine, baclofen, floxuridine	Pain management, hepatic arterial infusions for tumor site
Prometra II	2012	Morphine, baclofen	Severe spasticity, pain management

**Table 3:** FDA-approved intraocular IDDSs.

Implant	FDA Approval Date	Drug	Drug Class ^1^	Release Duration	Material ^2^	Indication
Vitrasert	1996	Ganciclovir	NSA	5–8 months	PVA, EVA	Cytomegalovirus retinitis
Retisert	2005	Fluocinolone acetonide	GC	2.5 years	PVA, Silicone	Noninfectious posterior uveitis, diabetic macular edema, central retinal vein occlusion
Ozurdex	2009	Dexamethasone	GC	6 months	PLGA	Retinal vein occlusion, uveitis, diabetic macular edema
Iluvien	2014	Fluocinolone acetonide	GC	3 years	PI	Diabetic macular edema, retinal vein occlusion
YUTIQ	2018	Fluocinolone acetonide	GC	3 years	PI	Posterior segment uveitis
Dextenza	2018	Dexamethasone	GC	1 month	PEG	Postoperative ocular inflammation, conjunctivitis, allergy
BIM Ring (Durysta)	2020	Bimatoprost	SAPG	6 months	Silicone, PP	Glaucoma
Susvimo	2021	Ranibizumab	a-VEGF MAB	6 months	PSu, Silicone	Neovascular age-related macular degeneration

Abbreviations: ^1^ NSA, Nucleoside analogue; GC, Glucocorticoid; a-VEGF MAB, anti-VEGF monoclonal antibody; ^2^ PVA, Polyvinyl alcohol; EVA, Ethylene Vinyl Acetate; PLGA, Poly-lactic-co-glycolic acid; PI, Polyimide; PEG, Polyethylenglycol; PSu, polysulphone; SAPG—structural analogue of prostaglandin F2α; PP—polypropylene.

**Table 4:** Some FDA-approved drug-eluting stents for coronary artery disease for coronary artery disease.

Implant	FDA Approval Date	Drug ^1^	Release Duration	Material ^2^	Biodegradability
Cypher	2003	SM	3 months	Stainless steel, PEVA/PBMA	Permanent
Taxus Express	2004	PTX	6 months	Stainless steel, SIBS	Permanent
Xience Alpine	2014	ELM	4 months	CoCr, PVDF-HFP	Permanent
Resolute Integrity	2012	ZLM	6 months	CoNi, BioLinx	Permanent
Orsiro	2019	SM	4 months	CoCr, PLLA	Coating biodegrades after 15 months
Synergy	2015	ELM	3 months	PtCr, PLGA	Coating biodegrades after 4 months
Absorb GT1 Bioresorbable Vascular Scaffold System	2016	ELM	3 months	PLLA, Pt markers	Stent degrades after >24 months

Abbreviations: ^1^ SM, Sirolimus; PTX, Paclitaxel; ELM, Everolimus; ZLM, Zotarolimus; ^2^ PEVA, polyethylene-co-vinyl acetate; PBMA, poly n-butyl methacrylate; SIBS, poly(styrene-b-isobutylene-b-styrene); PVDF-HFP, polyvinylidene fluoride hexafluoropropylene; BioLinx, BioLinx polymer; PLLA, poly (L-lactic acid); PLGA, poly(d,l-lactic-co-glycolic acid).

**Table 5:** Overview of the clinically approved (FDA) IDDSs discussed in the current study.

IDDS	Biodegradability/ Bio-Erodibility	Requires Removal?	Type of Implant
Norplant	No	Yes	Reservoir implant in PDMS tubing
Jadelle	No	Yes	Reservoir in PDMS core with silicone sheath
Implanon/ Nexplanon	No	Yes	Reservoir in EVA core and sheath
Testopel	Yes	No	Matrix in PVP
Viadur	No	Yes	Titanium cylinder with osmotically driven drug release (osmotic pump)
Vantas/ Supprelin LA	No	Yes	Reservoir in EVA
Probuphine	No	Yes	Matrix in EVA
Infusaid pump	No	Yes	Infusion pump with gas-mediated constant release
SynchroMed (SynchroMed II)	No	Yes	Peristaltic pumps
Codman 3000 (Intera 3000)	No	Yes	Infusion pumps without battery
Prometra II	No	Yes	Infusion pumps with battery
Vitrasert/ Retisert	No	Yes	Reservoir in drug pellet in PVOH/ EVA coating
Ozurdex	Yes	No	Matrix in PLGA
Iluvien/ YUTIQ	No	No	Reservoir in PVOH core in polyimide sheath
Dextenza	Yes	No	PEG hydrogel matrix
Susvimo	No	No	Refillable permanent reservoir system
BIM Ring (Durysta)–to complete description	Yes	No	Injectable polymer matrix
Gliadel	Yes (slow rate)	No	Drug eluting polymer matrix
Cypher	No	No	Stent with permanent DEPC
Taxus Express	No	No	Stent with permanent DEPC
Xience Alpine	No	No	Stent with permanent DEPC
Resolute Integrity	No	No	Stent with permanent DEPC
Orsiro	Coating biodegrades after 15 months	No	Stent with biodegradable DEPC
Synergy	Coating biodegrades after 4 months	No	Stent with biodegradable DEPC
Absorb GT1 Bioresorbable Vascular Scaffold System	Stent degrades after >24 months	No	Stent with biodegradable DEPC

Abbreviations: ^1^ PDMS, poly(dimethyl siloxane); EVA, ethylene vinyl acetate; PVP, polyvinylpyrrolidone; PVOH, Poly(vinyl alcohol); PEG, polyethylene glycol; DEPC, drug-eluting polymer coating.

**Table 6:** Highlights of clinical manifestations of IDDSs biointegration issues related to FBR.

Type of IDDSs	Reported Biointegration Issues that May Be Linked to FBR	References
Subcutaneous	Implant migration	[43]
	Difficulty with implant removal due to peri-implant scarring	[36]
	Implantation site aseptic inflammation or implant rejection	[51]
Infusion pumps as IDDSs	Inflammatory and dystrophic changes in spinal cord	[34]
	Fibrotic transformation of the skin pocket around metallic reservoir of the pump can affect the integrity of the reservoir–catheter connection and result in skin pocket fill with high doses of the drug	[67]
Ocular IDDSs	Poststeroid cataracts	[128]
Intracerebral IDDS (Gliadel)	Peri-implant inflammation	[116]
Drug-eluting Stents	Proliferation of neointima and thrombogenic complications (successfully prevented in modern IDDSs)	[125]
